# fMRI Evidence for a Dual Process Account of the Speed-Accuracy Tradeoff in Decision-Making

**DOI:** 10.1371/journal.pone.0002635

**Published:** 2008-07-09

**Authors:** Jason Ivanoff, Philip Branning, René Marois

**Affiliations:** Department of Psychology, Center for Integrative and Cognitive Neuroscience, Vanderbilt University, Nashville, Tennessee; New York University, United States of America

## Abstract

**Background:**

The speed and accuracy of decision-making have a well-known trading relationship: hasty decisions are more prone to errors while careful, accurate judgments take more time. Despite the pervasiveness of this speed-accuracy trade-off (SAT) in decision-making, its neural basis is still unknown.

**Methodology/Principal Findings:**

Using functional magnetic resonance imaging (fMRI) we show that emphasizing the speed of a perceptual decision at the expense of its accuracy lowers the amount of evidence-related activity in lateral prefrontal cortex. Moreover, this speed-accuracy difference in lateral prefrontal cortex activity correlates with the speed-accuracy difference in the decision criterion metric of signal detection theory. We also show that the same instructions increase baseline activity in a dorso-medial cortical area involved in the internal generation of actions.

**Conclusions/Significance:**

These findings suggest that the SAT is neurally implemented by modulating not only the amount of externally-derived sensory evidence used to make a decision, but also the internal urge to make a response. We propose that these processes combine to control the temporal dynamics of the speed-accuracy trade-off in decision-making.

## Introduction

A fundamental problem faced by any decision maker is finding a suitable compromise between making quick and yet accurate decisions. Accurate decisions can be achieved by accumulating as much information as possible at the expense of the additional time spent on the decision-making process. The alternative approach is to make fast decisions, but at the increased risk of making errors. These two approaches are governed by a well-known trading relationship between speed and accuracy performance measures, the speed-accuracy tradeoff (SAT) [1]–[4]. The SAT is a ubiquitous property of decision-making agents, found not only in humans, but also in a wide range of animal behaviors, ranging from odor discrimination in rats [5], to foraging in honeybees [6] and nest hunting in ants [7].

**Figure 1:**
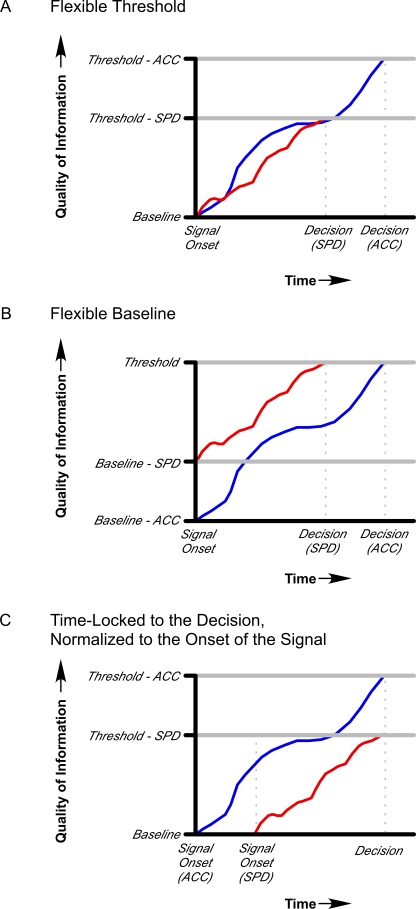
An illustration of a simple accumulator model of the speed-accuracy tradeoff (SAT). According to this type of model, task-relevant evidence (e.g. motion coherence) accumulates over time from a starting point until a threshold for decision is reached (horizontal grey bars). The vertical stippled lines mark the time, along the x-axis, when a response would be made. (a) In a flexible threshold account of the SAT, emphasizing the speed (SPD) of responding lowers the threshold for decision-making relative to emphasizing accuracy (ACC) of responding, thus reducing the amount of accumulated evidence (and time) prior to the response. (b) In a flexible baseline account of the SAT, emphasizing the speed of responding would increase the baseline level of activity towards a decision threshold, thereby reducing the amount of evidence, and hence the time, that is required to reach that threshold. These two models are not necessarily mutually exclusive. (c) If the accumulation functions are aligned to the time that the decision is made, and normalized to onset of the signal, both the flexible threshold and flexible baseline accounts predict less accumulated signal-based evidence at the time of the decision when response speed is stressed.

Despite the fact that the SAT reveals a pervasive and fundamental constraint in information processing, its neurobiological underpinnings are not yet understood. Great progress has been made in our understanding of the neural basis of decision-making in both monkeys and humans [8]–[30] but many of these studies have not specifically addressed the mechanism by which the SAT is neurally implemented. By contrast, several computational models of decision-making, particularly random walk and accumulator models, explicitly incorporate a theoretical framework for SATs [31]–[34]. While the models differ according to the specific dynamics of information accrual, they generally share the common feature that sensory evidence accumulates over time from some baseline level to a decision threshold. Such models have been successful in accounting for the behavioral performance in a wide range of decision-making tasks [32], and have received considerable support from neurophysiological studies demonstrating accumulation of neural activity towards a decision threshold across several brain regions [10]–[12], [16], [17], [20], [22], [23], [26], [28]–[30], [35]–[37]. Within this framework, instructions that emphasize speed of a task are modeled as a lowering of the decision threshold compared to instructions that emphasize the accuracy of performance [32], [38]–[41] (**Figure 1a**). Lowering the decision threshold reduces the amount of evidence accumulated prior to the decision, thereby leading to more frequent errors. Alternatively, the SAT may be modeled by shifting the starting point (baseline) of information accrual towards a decision boundary while maintaining a fixed threshold. In some models threshold-shifts are equivalent to shifting the starting point [42] (**Figure 1b**). There is currently little behavioral or neurobiological evidence to support one implementation of the SAT over another [38], [43], [44] (e.g., see **Figure 1c**). While trial-by-trial variations in RTs may be explained by differences in rates of neural information accumulation to a fixed threshold [12], [43], these results do not necessarily speak to the neural mechanism by which speed-accuracy instructions are implemented in the brain [45].

**Figure 2:**
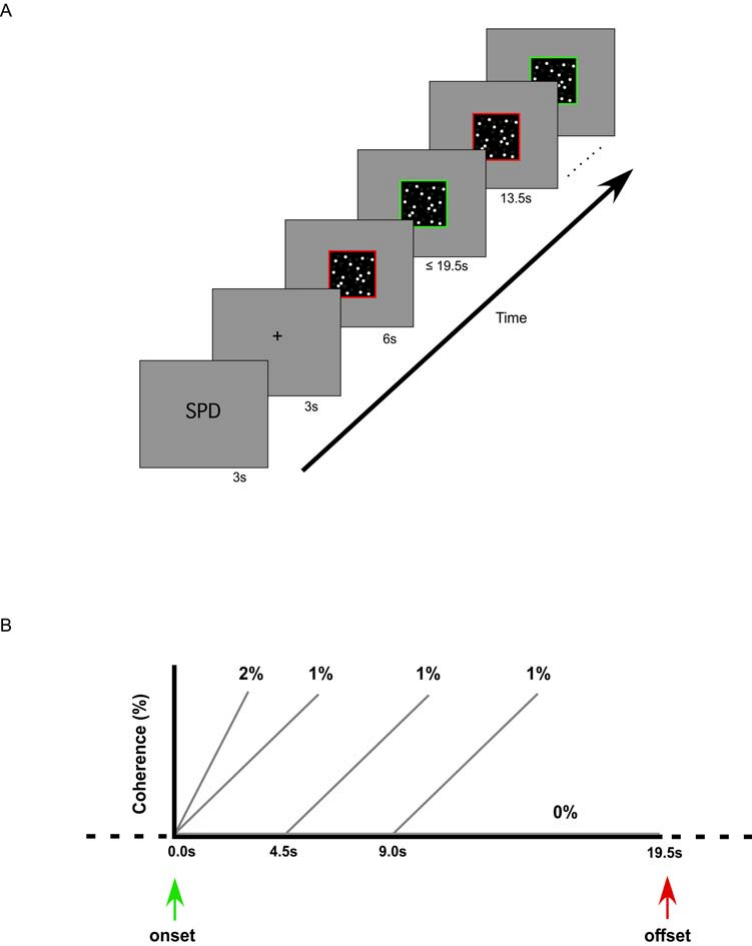
(a) Trial sequence. A cue (SPD or ACC) instructed participants to heed the speed or accuracy of their decisions at the onset of a block of seven trials. A red-framed display containing randomly moving dots appeared 3 s later. Trial onset was signaled by the frame changing from red to green. Once a response was made, either within the 19.5 s trial duration or at the end of the trial, the frame returned to red until the next trial. (b) Trial types. ′Coherence′ trials consisted of a 1%/s rise in motion coherence that began either 0 s, 4.5 s, or 9 s following trial onset. ′Baseline′ trials contained no motion coherence (0%) throughout the trial. A third trial type included a 2%/s rise in coherence that began at trial onset.

Using fMRI, the present study aimed at identifying the neural mechanisms by which SAT is implemented in a simple decision-making task. To do so, we used a modified version of a motion discrimination task (**Figure 2**) that has been used extensively in psychophysical and neurophysiological studies of decision-making [30], [46]–[48]. This task involved detecting and judging the direction of motion coherence in a dynamic random-dot display while emphasizing either the speed or accuracy of the decision. Importantly, unlike previous neurophysiological and psychophysical experiments in which motion coherence appears abruptly and transiently, in the current study the proportion of dots moving coherently was gradually increased during the course of a trial. Thus, relative to a procedure with an abrupt onset of constant motion coherence, the gradated approach slowed the decision-making process to the extent that it was temporally resolvable with fMRI. We also included trials without motion coherence, as these trials assessed how the speed-accuracy instructions influenced baseline levels of activity in the same brain regions that responded to motion coherence information.

## Results

### Localizer Task

The brain regions probed in the SAT experiment were first identified using a localizer task designed to isolate areas involved in all major stages of information processing of the motion direction discrimination task, from motion perception to decision-making and motor response. We hypothesized that the SAT instructions should modulate the activity of a subset of the brain regions along this information processing pathway. In a blocked-trial design, we contrasted brain activity obtained while participants made a motion discrimination response to a dynamic dot display containing 60% motion coherence with brain activity acquired while subjects passively viewed a static version of the dot display. We observed activation in a large network of areas including sensory cortex (extrastriate cortex, MT^+^), fronto-parietal association cortex (supplementary motor area [SMA], pre-supplementary motor area [pre-SMA], motor and premotor cortex, insula, anterior cingulate cortex [ACC], lateral frontal/prefrontal cortex, anterior parietal cortex), and sub-cortical foci (thalamus, putamen, and cerebellum) (**Table 1**). The primary motor cortex (M1) was further distinguished from other motor cortical areas as the region of precentral gyrus that responded more to contralateral than ipsilateral manual responses in the event-related fMRI experiment (see below, Methods). Together, these areas served as group-defined regions of interest (ROIs) for the event-related SAT experiment.

**Table 1:** Brain Regions Identified with the Localizer Task.

		Left				Right			
Region	B.A.	x	y	z	voxels	x	y	z	voxels
Superior Frontal Gyrus	6	−18	−11	67	945				
Precentral Gyrus	6	−24	−16	63	551	32	−17	59	490
Precentral Sulcus	6	−29	−8	61	205				
S1 and M1	3,4	−35	−24	55	911	37	−24	56	679
Pre-SMA	6	−2	5	52	259	4	7	56	670
SMA	6	−2	−5	55	592	3	−4	56	583
Anterior Cingulate	24	−3	0	38	714	4	0	38	700
Anterior Cingulate	32	−2	15	36	678	3	15	36	694
Anterior IPS	40					43	−46	54	107
DIPSA	40	−43	−30	51	546	47	−33	51	812
dPM	6	−28	−9	49	890	29	−9	47	1472
Postcentral Gyrus	1,2,3	−53	−17	42	1892	50	−20	42	1327
	1,2,3	−54	−18	24	2343	52	−18	24	1656
vIPL	2,40	−39	−32	41	738	37	−30	42	1872
vPM	6,9	−51	−1	39	68	50	5	39	119
pLPFC	9,44,45	−55	8	22	312	53	11	26	442
TPJ	40	−52	−27	24	999	53	−32	26	1998
Thalamus		−11	−14	10	1505	12	−17	11	2269
Insula	13	−41	−2	5	2876	40	1	6	3040
Putamen		−21	7	2	3587	19	7	4	3617
Extrastriate	17,18,19	−21	−88	1	1333	24	−88	3	3322
Superior Colliculus		0	−27	2	343				
Anterior Insula	13	−40	10	1	2139	37	15	2	2952
MT+	19,21,37	−32	−64	−7	715	43	−64	1	3327
Anterior IFG	46	−34	30	0	132				
Anterior Cerebellum		−8	−58	−8	1761	5	−58	−8	2242
Posterior Cerebellum		−21	−57	−19	2366	17	−57	−19	1651

Notes:

x,y,z co-ordinates according to the Talairach and Tournoux atlas [93].

B.A. Brodmann Area; M1 = Primary motor cortex; Pre-SMA = Pre- Supplementary Motor Area; SMA = Supplementary Motor Area; IPS = Intraparietal sulcus; DIPSA = dorsal IPS anterior; vIPL = ventral inferior parietal lobe; IFJ = Inferior frontal junction; pLPFC = posterior lateral prefrontal cortex; TPJ = temporo-parietal junction; IFG = Inferior frontal gyrus; dPM = dorsal premotor cortex; vPM = ventral premotor cortex.

### The SAT Task

The task was designed to make the neural effects of the SAT temporally resolvable with fMRI by gradually increasing motion coherence during a trial. This ‘gradated fMRI’ approach has previously been successful in dissociating other aspects of visual information processing [49], [50]. There were three trial types in the present experiment (**Figure 2**). Most trials (42%), hereafter named ‘coherence’ trials, included a 1% motion coherence increase per second, with the onset of coherence beginning at different times (0 sec, 4.5 sec or 9 sec from trial onset) during the course of the trial in order to prevent subjects from predicting the onset of motion coherence. The other trials were evenly divided (29% each) between ‘baseline’ trials, in which motion remained random (0% coherence) throughout the trial, and trials in which motion coherence rose quickly (2%/s coherence rate) at trial onset. The baseline trials were included to assess baseline activity in the absence of any motion coherence, while the 2%/s trials served to ensure that subjects were attending to motion coherence of the display from trial onset. At the beginning of separate blocks of trials, participants were instructed to emphasize either the speed or accuracy of task performance (see Methods).

### Behavioral Results

**Figure 3:**
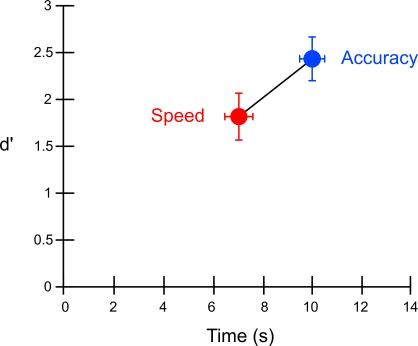
Sensitivity to detect coherent motion (d') versus response time (s) as a function of Speed and Accuracy instructions.

As expected, there was a tradeoff in performance between response speed and accuracy. Specifically, while motion detection was better in the accuracy condition [*t*(12) = 2.65, *p*<0.05], reaction times were shorter in the speed condition [*t*(12) = 5.61, *p*<0.0005] (**Figure 3**) [40]. Importantly, subjects adopted a more liberal decision criterion in the speed than the accuracy condition (*c*_speed_ = −.71, *c*_accuracy_ = .05; [*t*(12) = 7.83, p<0.0001]) (see Methods). Thus, decisions were faster and based upon less sensory evidence in the speed condition than in the accuracy condition.

### fMRI Results I. Simulus-locked

**Figure 4:**
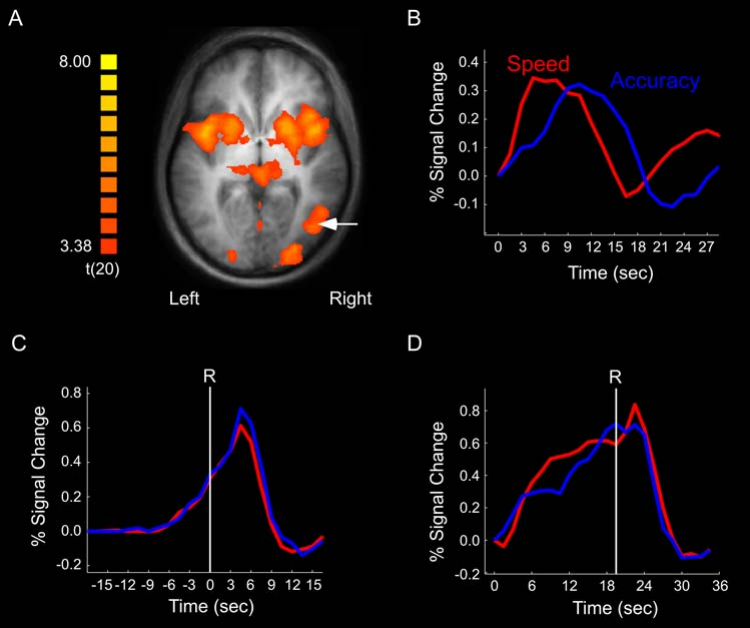
fMRI results for MT+. (a) SPM of right MT+ (white arrow) in localizer experiment. (b) Speed and accuracy time courses for the coherence trials, time-locked and normalized to the onset of stimulus coherence. (c) Speed and accuracy time courses for the coherence trials, time-locked to the onset of the response (R) and normalized to the onset of stimulus coherence. (d) Speed and accuracy time courses for the baseline trials, time-locked and normalized to the onset of the trial.

Hemodynamic responses were time-locked to the onset of motion coherence within each trial, and percent signal change was calculated with reference to the volume acquired at the onset of motion coherence (i.e., normalization). These stimulus-locked time courses revealed distinct activation peaks for speed and accuracy conditions across all ROIs (see **Figure 4b** for the Right MT^+^). The mean peak latency difference between the speed and accuracy conditions in MT^+^ corresponded well to the mean RT difference between these conditions across subjects (3.23 s +/− 1.28 SEM, and 2.97 s +/− .53 SEM, respectively, *r* = .71, *df* = 12, *p*<.01). However, further interpretation of the stimulus-locked data is challenging as the amplitude and width of these time courses are temporally blurred by the variability in response times across trials and subjects. Hence, in order to isolate the neural processes underlying the SAT in decision-making, we turned to a response-locked approach.

### fMRI Results II: Response-locked

#### Coherence trials

According to computational models and neurophysiological studies [10]–[12], [16], [20], [22], [23], [28]–[36], the activity in brain regions involved in decision-making should increase from stimulus coherence onset up to the decision threshold. Moreover, activity at the time of the decision ought to be higher following instructions to emphasize accuracy than following instructions to emphasize response speed, reflecting the greater accumulation of evidence in the former condition. To identify brain regions exhibiting a surge in activity from coherence onset up to the time of the decision, we time-locked the hemodynamic time courses to the onset of the response and normalized to the onset of the motion coherence. Given that response time should closely follow the time of the decision [20], [28], activity around the time of the response should correspond to the decision threshold, whereas activity following response onset would include response-related activity. Thus, the hemodynamic activity average of volumes acquired at and immediately prior to response time was considered to represent activity near decision time (see Methods).

**Figure 5:**
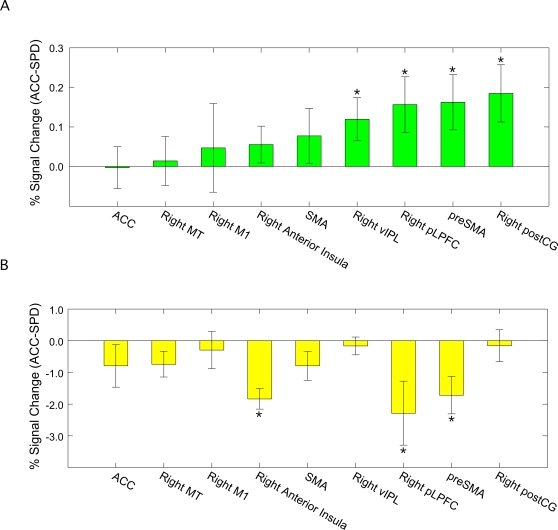
Activation differences between accuracy and speed conditions at response time (i.e., the average of the pre-response and response volumes) for sensory, motor, and premotor ROIS (see Table 1 for complete ROI list). (a) ′ Coherence ′ and (b) ′ Baseline ′ conditions. Error bars reflect the SEM of the difference. *: p<0.05. Although the right ventral inferior parietal lobe (vIPL) and postcentral gyrus ROIs in (a) and the right anterior insular ROI in (b) showed significant activation differences (accuracy vs. speed), these brain regions did not demonstrate significant activity increase above baseline in both accuracy and speed conditions (see Tables S1 and S2).

Of the several ROIs probed, only a few showed significant activity in both the speed and accuracy conditions, and even fewer showed differential activity across these two conditions (**Figure 5**; see also **Table S1**). Sensory cortex (MT^+^) exhibited activity increase prior to the response under both speed and accuracy instructions (**Figure 4c**). Importantly, this activity was indistinguishable between speed and accuracy conditions at the time of the response. This finding was replicated even when we only probed the maximally activated voxel of individually defined ROIs, suggesting that these results are not due to low sensitivity of the group-averaged ROI analysis. The data therefore indicate that the build-up of activity in MT^+^ is not differentially affected by the SAT.

**Figure 6:**
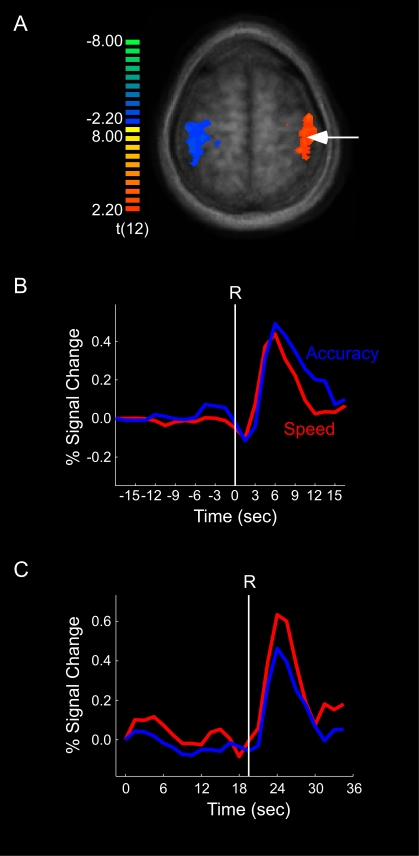
fMRI results for right primary motor cortex (M1). (a) Isolation of M1 (left) with the left-right manual response contrast of the event-related experiment. White arrow indicates Right M1. (b) Speed and accuracy time courses for the coherence trials, time-locked to the onset of the response (R) and normalized to the onset of stimulus coherence. (c) Speed and accuracy time courses for the baseline trials, time-locked and normalized to the onset of the trial.

In contrast to sensory cortex, the primary motor cortex failed to demonstrate any increase in activity under speed or accuracy conditions (**Figure 6b**). Instead, activity was restricted to post-response volumes. These results suggest that the primary motor cortex is not directly involved in the decision-making process, and that its role in the current task is purely motoric. Little or no increase in activity was also observed in several other brain regions, including the anterior cingulate cortex (see **Table S1**).

**Figure 7:**
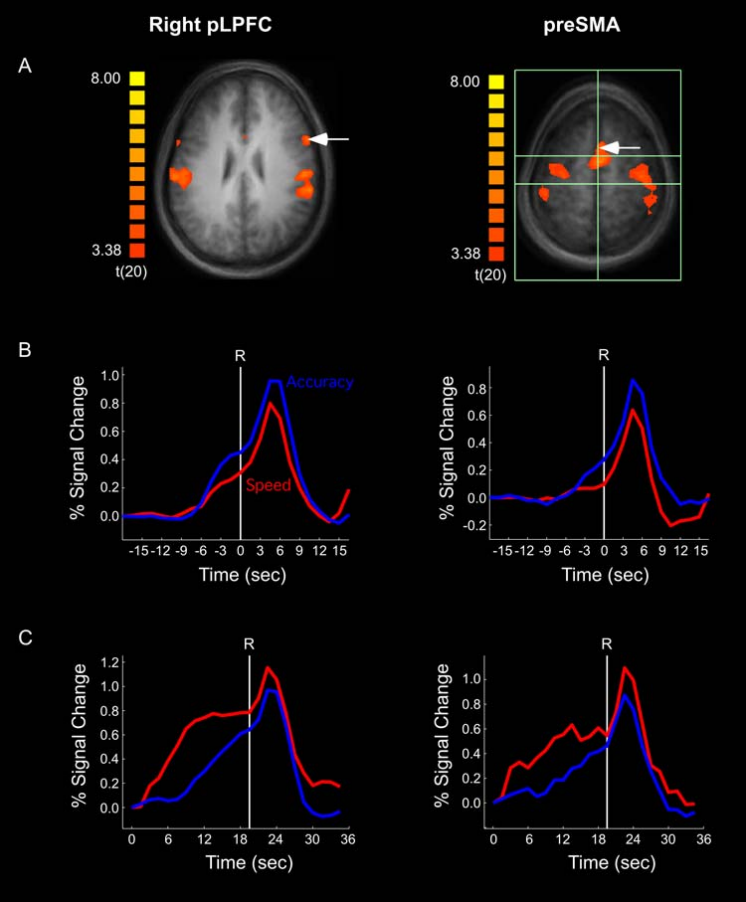
fMRI results for pLPFC (left column) and the average of left and right pre-SMA (right column): (a) Location of right pLPFC (white arrow) and pre-SMA (white arrow) in the localizer task. The anterior and posterior horizontal bars in the pre-SMA figure indicate the coronal planes of the anterior and posterior commissures. (b) Speed and accuracy time courses for the coherence trials, time-locked to the onset of the response (R) and normalized to the onset of stimulus coherence. (c) Speed and accuracy time courses for the baseline trials, time-locked and normalized to the onset of the trial.

Unlike sensory (MT^+^) and primary motor cortex (M1), the activity of neural regions in medial frontal and lateral prefrontal cortex increased prior to the response under both speed and accuracy instructions. Moreover, some of these regions were also differentially sensitive to speed-accuracy instructions. Specifically, the activity build-up in a region of the posterior lateral prefrontal cortex (pLPFC) extending into anterior premotor cortex (**Figure 7b**, left), and in the pre-SMA bilaterally **(** **Figure 7b**, right), was greater in the accuracy condition than in the speed condition (*ps*<.05). These results are not only consistent with accumulator models of decision-making [32], [39], [40], [51], but also with neurophysiological evidence that accumulation of activity occurs in ‘central’ stages rather than in early sensory or late motor stages of information processing [16], [20], [26], [30], [52].

#### Baseline trials

Emphasizing the speed of the decision may also increase baseline activity (see **Figure 1b**). We investigated this possibility by measuring the effect of speed-accuracy instructions on brain activity in the ‘baseline’ (0% motion coherence) trials. The primary motor cortex did not demonstrate any ramping of activity in these trials (**Figure 6c**). By contrast, the activity of sensory cortex (MT^+^) increased during the baseline trials (**Figure 4d**), with a non-significant trend for a greater build-up in the speed than the accuracy conditions (*p* = 0.09). More importantly, right pLPFC and pre-SMA not only showed ramping of activity up to the time of the response (**Figure 7c**), but also greater activity under speed than under accuracy conditions [right pLPFC: *t*(12) = 2.27 , *p*<0.05; pre-SMA: *t*(12) = 2.92, *p*<0.05], an activity pattern that is opposite to that obtained during coherence trials in these two brain regions.

These results indicate that speed-accuracy instructions modulate baseline activity in a specific subset of brain regions (see also **Table S2**). Furthermore, the results generally mirror those observed in the coherence trials: M1 does not exhibit pre-response build-up of activity, MT^+^ is relatively insensitive to speed-accuracy instructions, and the frontal regions (pLPFC and pre-SMA) are highly sensitive to speed-accuracy instructions, albeit in diametrically opposite ways during coherence and baseline trials.

### Flexible Threshold vs. Flexible Baseline Accounts of the SAT

**Figure 8:**
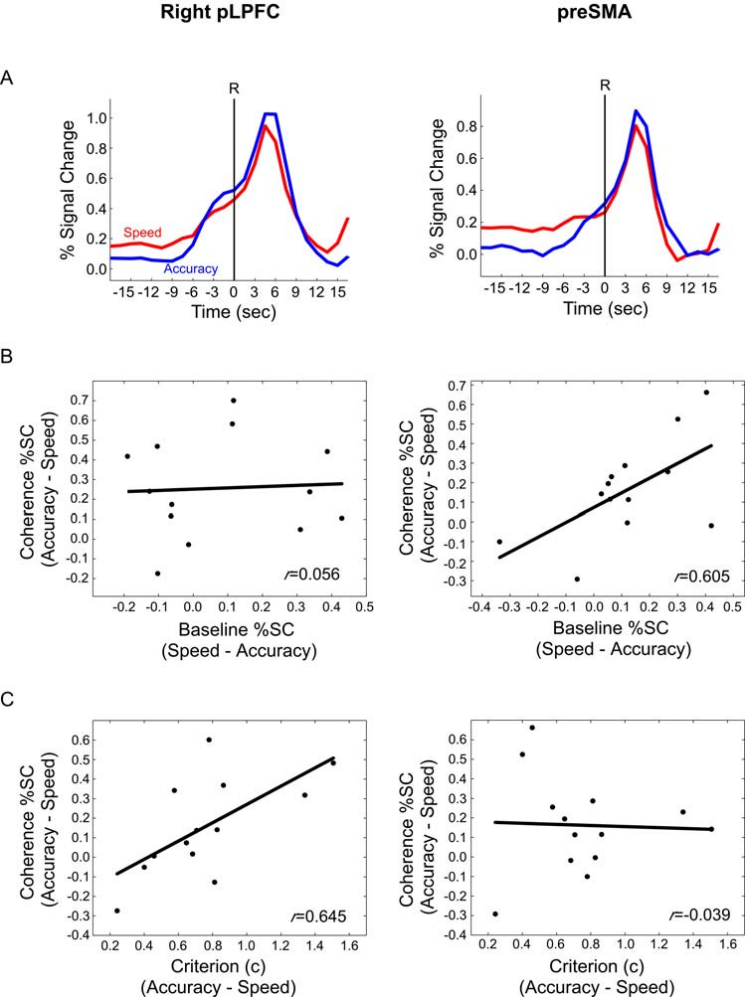
fMRI results for right pLPFC (left column) and average pre-SMA (right column). (a) Speed and accuracy time courses for the coherence+baseline trials, time-locked to the onset of the response, and normalized to the % signal change in the baseline trials. (b) Linear regression plots between the accuracy-speed differences in activity in the coherence and baseline trials. (c) Linear regression plots between the accuracy-speed difference in the decision criterion (c) and the accuracy-speed difference in activity for the coherence trials.

The inverse effects of speed and accuracy instructions on coherence and baseline trial activity in pLPFC and pre-SMA are compatible with a flexible baseline account of the SAT. According to this account, the amount of evidence-related activity that needs to be accumulated to a decision threshold is determined by its starting point (see **Figure 1b**): the higher the starting point (baseline activity), the less evidence needs to be accumulated to reach a fixed decision threshold. This account predicts that the activity difference between speed and accuracy in the baseline trials should be matched by an equal but inverse activity difference between accuracy and speed in the coherence trials. To test this prediction, we assessed whether the sum of the baseline-related and coherence-related activity would be equivalent in speed and accuracy conditions (see Methods). The results of this “coherence+baseline” analysis effectively revealed that activity levels around the time of the response are not different between speed and accuracy conditions (*p*s>.40) in both pLPFC and pre-SMA (**Figure 8a**). Importantly, this result should not only hold for group-averaged data, but for individual subject's data as well. Specifically, an individual who demonstrates a large speed-accuracy difference in baseline activity should exhibit an equally large accuracy-speed difference in coherence-related activity (in order to arrive to a fixed threshold). A significant correlation was observed in pre-SMA [*r* = .605, *df* = 12, *p*<0.05] (**Figure 8b**), but not in pLPFC [*r* = .056, *df* = 12, *p*>0.85]. These results strongly suggest that speed-accuracy instructions modulate baseline activity in pre-SMA, thereby modifying the amount of evidence that must be accrued prior to reaching a decision threshold.

In contrast to the pre-SMA, the correlational analysis of pLPFC activity was not consistent with a flexible baseline model of the SAT. To determine whether pLPFC function may instead be more consistent with a decision threshold account of the SAT, we examined whether its activity tracks a behavioral measure of decision performance, the decision criterion (*c*) of signal detection theory (see Methods). As noted above, this criterion metric varied with speed-accuracy instructions. Specifically, we measured the correlation between the accuracy-speed differences in *c* with the accuracy-speed differences in hemodynamic activity during the coherence trials. Remarkably, this correlation was significant in pLPFC [*r* = .645, *df* = 12, *p*<0.05], but not in pre-SMA [*r* = −.039, *df* = 12, *p*<0.90] (**Figure 8c**) or in any other ROI (*p*'s>0.16). These results suggest that the pLPFC activity at the time of the response reflects differences in individual subjects' decision criterion due to speed-accuracy instructions. Put another way, how much an individual subject's decision criterion is modified by speed-accuracy instructions dictate how much coherence-related activity is accumulated in pLPFC (but not in preSMA).

Additional evidence that the pLPFC and preSMA make different contributions to decision-making come from the analysis of false alarm trials (i.e., trials in which subjects made an early response when there was 0% motion coherence at the time of that response). Brain regions that accumulate information ought to have greater activity in the growing presence of signal (hits) than in the absence of signal (false alarms). Correspondingly, in pLPFC, the activation for hits was greater than it was for false alarms [*t*(11) = 3.17, *p*<0.01] (**Figure S1a**). In stark contrast, hits and false alarms activated the pre-SMA comparably (*p*>0.25; **Figure S1b**), suggesting that false alarms may primarily arise from changes in internal (baseline) levels of activity rather than by fluctuations in sensory evidence (see Discussion).

## Discussion

The principal finding of the present study is that the SAT in decision-making may be neurally implemented by more than one mechanism. Although several computational models posit that the SAT arises from changes in the decision boundary [38], [41], [42], [44], [45], [53], [54], a mathematically equivalent outcome can be obtained from a baseline-shift [42] (see **Figure 1**). As elaborated below, our results suggest that both processes may account for the SAT. Specifically, the speed-accuracy trade-off may arise from both threshold- and baseline-shifts, implemented by distinct areas of the prefrontal cortex (pLPFC) and medial frontal cortex (pre-SMA). Importantly, these effects were not observed in either sensory (MT^+^) or primary motor cortex, further suggesting that the SAT in decision-making is a property of a central, prefrontal network rather than arising from modulations at early sensory or late motor stages of processing [16], [17], [20], [26], [30], [52].

### Sensory and Motor Cortex

Although MT^+^ showed coherence-related ramping of activity prior to the response, such activity build-up was similar under both speed and accuracy conditions, suggesting that this sensory cortical area is not differentially sensitive to these instructional sets. Since motion coherence was about 2.8% higher at response in the accuracy than in the speed condition, one might have expected slightly higher activity in MT^+^ in the former condition. However, given that the hemodynamic response of this brain region is weakly modulated by low levels of coherent motion [55], it is not surprising that MT^+^ was relatively insensitive to the small difference in motion coherence between speed and accuracy conditions in the present experiment. In addition to being driven by bottom-up sensory stimulation, MT^+^ is also sensitive to top-down modulation [56] as evidenced by the build-up of activity in the baseline trials, though this buildup was generally impervious to SAT instructions. The insensitivity of MT^+^ to the speed-accuracy manipulation, particularly during the coherence trials, strongly implies that this brain region does not encode the observer's decision, consistent with neurophysiological studies indicating that MT^+^ neurons supply sensory evidence to other brain regions that accumulate the evidence towards a decision threshold [20], [43]. In this context, the role of visual cortex may primarily consist in representing the sensory information for accurate stimulus identification [8], with the accumulation of such information taking place in central, decision-making areas of the brain [30].

In contrast to sensory cortex, the primary motor cortex (M1) showed no evidence of build-up of activity prior to the response in either the coherence or baseline trials, suggesting that it is not part of the decision-making network [26], [52]. Rather, M1 likely occupies a stage of information processing downstream from decision-making, most likely corresponding to response execution. Like M1, the anterior cingulate cortex (ACC) was only activated after the response. Unlike M1 however, an extensive literature suggests that the post-decisional activity in ACC is probably associated with performance monitoring and/or adjustment [57]–[59], rather than response execution *per se*. Taken together, these results suggest that the neural manifestations of the SAT are not widespread throughout the brain, sparing sensory, motor and some higher association areas. Rather, the SAT appears to be implemented in a specific subset of premotor and prefrontal areas.

### Modulation of frontal/prefrontal activity by the SAT

Speed-accuracy instructions differentially modulated activity in two regions of frontal/prefrontal cortex (pLPFC and pre-SMA), a finding consistent with the notion that the frontal lobe is critical for cognitive control, decision-making, and response selection [13], [15], [17], [28], [38], [57], [60]–[71]. These results do not rule out the possibility that additional brain regions, which showed non-significant trends in the present experiment (e.g. parietal ROIs, **Tables S1 and S2**), may be critically involved in the SAT of decision-making under different task contexts (e.g. oculo-motor tasks [15], [72]). Nevertheless, the key findings of the present study should reveal general insights into the neural mechanisms of SAT that extend beyond the specific sensori-motor task used in the present experiment given that these lateral and dorsal premotor regions are neither strictly sensory nor motor [64], [73].

Speed-accuracy instructions generally affected activity within the pLPFC and pre-SMA in a similar manner. There was greater pre-response activity with the accuracy condition than there was with the speed condition when the motion coherence signal was present (coherence trials). Strikingly, the reverse was true when the signal was absent (baseline trials): activity was greater in the speed than in the accuracy condition in both brain regions. However, further analyses revealed that speed-accuracy instructions differentially affected neural processing in pLPFC and pre-SMA.

The pre-SMA demonstrated a gradual ramping of baseline activity prior to the response. Importantly, this increase in activity, which occurred throughout the duration of baseline trials, was not present in the primary motor cortex, suggesting that it does not reflect a late form of motor preparation. On the other hand, this activity build-up was not unique to the pre-SMA, as it was present in several other brain regions, including MT^+^ and pLPFC. Unlike these other areas, however, in pre-SMA the activity difference between accuracy and speed conditions during baseline trials was inversely correlated with the activity difference between accuracy and speed conditions on coherence trials (**Figure 8b**). These results are highly consistent with a baseline account of the SAT (**Figure 1**) in which speed-accuracy instructions affect the point where evidence begins to accumulate to a fixed threshold: the higher the starting point (i.e., greater baseline activity), the less evidence is necessary (lower coherence-related activity) to reach the decision threshold. Consistent with a flexible baseline account of pre-SMA activity, the medial frontal cortex has been shown to exhibit anticipatory/preparatory activity ahead of motor responses [74]–[76], with such increases in baseline activity concomitant with decreases in reaction times [77], [78]. Interestingly, anticipatory processes might also account for the general ramping of activity we observed throughout the duration of baseline trials (see **Figures 4d** **and** **7c**), as the probability that subjects will need to make a response (given that they have yet to execute one; i.e. the hazard rate [77]) increases during the course of a trial. Together, these neuroimaging and neurophysiologial findings converge with behavioral work suggesting that manipulating the urgency to respond by differentially emphasizing the speed or accuracy of a response is behaviorally tantamount to changing the likelihood of making a response [38]. This account is also compatible with the finding that hits and false alarms similarly activate the pre-SMA, as false alarms may have arisen from an excessive urgency to respond despite scant physical evidence for motion coherence (see **Figure S1b**).

In contrast to the pre-SMA, there was no correlation between baseline- and coherence-related activity in pLPFC. However, the pLPFC activation difference between speed and accuracy conditions during coherence trials did correlate with the difference between speed and accuracy in the decision criterion, *c*. Emphasizing the speed of responding decreases the decision criterion, thereby limiting the accrual of evidence before a response is made. Thus, the activity pattern in pLPFC is more consistent with a flexible-threshold than a flexible-baseline account of the SAT. Activity in pLPFC may therefore reflect the accumulation of coherence-related evidence – i.e. the integral of motion coherence signal over time - towards a decision criterion, as has been observed in single-cell studies [12], [16], [20], [30]. This hypothesis is further supported by the analysis of false alarms, as there was less activation in pLPFC when a decision was erroneously reached on trials without motion coherence than when a decision was correctly reached with motion coherence (see **Figure S1a**). This conclusion meshes nicely with the neurophysiology literature suggesting that the lateral prefrontal cortex plays a role in converting continuous accumulation of sensory evidence into a discrete decision code [17].

Alternative accounts of pLPFC activity are not supported by the data. The pLPFC activity is unlikely to encode absolute motion coherence levels, as the difference in coherence activity between the accuracy and speed conditions was not even discernable in the brain region (i.e., MT^+^) most sensitive to the type of motion coherence display used in the present experiment. Furthermore, ‘preparatory activity’ [78]–[80], general arousal, or time-on-task (trial duration) effects cannot easily account for the coherence-related activity in pLPFC given that they would not predict opposite patterns of activation for speed and accuracy conditions during coherence and baseline trials, and given that the pLPFC pattern of activity was not widespread across the brain. Nevertheless, that this area did show increased activity in baseline trials suggests that it is also susceptible to top-down input, consistent with single-cell work demonstrating the influence of both bottom-up (evidence-related) and top-down (anticipatory) factors in decision-making activity in the parietal and dorsolateral prefrontal cortex of macaques [17], [30]. However, because the accuracy-speed activity difference on baseline trials does not correlate with the accuracy-speed difference on coherence trials, we conclude that the SAT is primarily implemented in this brain region by a modulation of evidence-related activity rather than by changes in top-down activation.

Many computational models of decision-making implement the SAT as a change in the boundary separation between the starting (baseline) and end (threshold) points of accumulation of evidence [32], [39], [40], [51], [81]. Even though most models presume that it is the threshold that is modulated by speed-accuracy instructions, computationally similar results may be obtained with baseline shifts in detection tasks e.g., [42]. A central goal of the present study was to determine which, if any, of these two mechanisms is implemented in the brain. Our results suggest that both may underlie the SAT, with a baseline shift in pre-SMA and a decision threshold shift in pLPFC. Importantly, these two mechanisms are not incongruous with one another: a differential emphasis on the speed and accuracy of deciding may be neurally implemented in different manners in distinct brain regions if these brain regions provide unique contributions to the decision-making process. Indeed, our results are highly consistent with the notion that lateral and medial premotor/prefrontal cortices play unique roles in decision-making [82]–[85]. According to this proposal, the lateral premotor cortex is preferentially involved in externally (sensory) guided selection of movement, whereas the medial premotor cortex is primarily recruited for internally guided selection and preparation of movement. Consistent with this idea, several neuroimaging studies point to the pre-SMA as a brain region involved in internally-generated initiation and selection of movement [75], [86], [87], while others have observed lateral prefrontal and premotor activity during externally-guided selection of responses [15], [62], [73], [88]–[90]. Indeed, the different sensitivities of lateral and medial frontal cortex to external and internal cues for movement selection offer a framework that can account for the distinct pLPFC and pre-SMA responses observed in the SAT. Within this framework, speed-accuracy instructions have at least two effects: a modulation of the internal urge to make a response, brought about by changes in baseline activity in medial frontal cortex (pre-SMA), and a modulation of the amount of sensory evidence required to reach a decision, brought about by changes in pLPFC activity. The advantage of this dual process account is that it may ensure that perceptual decision-making is jointly based on information about the environment and the internal state of the decision maker. Indeed, it likely is a combination of externally and internally guided factors, and the interaction between brain regions processing these factors, that ultimately determines if, and especially when, we reach a decision.

## Methods

### Localizer Task

#### Subjects

Twenty-one subjects (ages 20–31, 7 females) participated in the study for pay after signing an informed consent document. The Vanderbilt University Institutional Review Board approved the study protocol.

#### Task design and procedure

The localizer task included two conditions, each presented in 30 s blocks of 10 trials. In the static display condition, a trial consisted of 1.5 s of a black fixation cross on a grey background replaced for 1.5 s by the presentation at fixation of a 3°×3° black square containing 100 randomly scattered white pixels (dots). Participants were instructed to simply attend to the display. In the motion coherence condition, the trial was identical except that the static display was substituted by a dynamic display in which 60% of the 100 dots moved in a coherent direction, either leftward or rightward, with the remaining 40% of dots moving in random directions. Dots that exited the frame were replaced by dots on the opposite side. The dots moved at a rate of 1.12°/s. Participants were instructed to respond quickly and accurately about the direction of coherent motion by pressing one of two buttons with the left and right index fingers. There were six 30 s blocks of each of the two conditions per fMRI run, and one run of the localizer task per fMRI session.

The localizer and slow event-related tasks (see below) were programmed in MATLAB (Mathworks, Natick, MA) with the Psychophysics Toolbox extension [91], [92], and were presented using an iMAC. The visual display was presented on an LCD panel and back-projected onto a screen positioned at the front of the scanner. Subjects lay supine in the scanner and viewed the display on a mirror positioned above them. Responses were acquired with two MRI compatible button boxes (Rowland Institute of Science, Cambridge, MA), one for each index finger.

#### fMRI data acquisition and analysis

Imaging data was acquired with a 3 Tesla Philips Intera Achieva scanner at the Vanderbilt University Institute of Imaging Science. T1 2D and 3D anatomical images were acquired using standard parameters. T2* image parameters were: 1.5 s TR, 35 ms TE, 70° flip angle, with a field of view of 24 cm^2^ and 128×128 matrix size, and 25 five-mm thick slices (1.875 mm^2^ in-plane) with no gap. Brainvoyager QX (v1.4; Brain Innovation, Maastricht, Netherlands) was used to preprocess the data and generate statistical parameter maps. The fMRI data was motion corrected, slice scan time corrected, corrected for linear drift, spatially smoothed at 4 mm, and scaled to the Talairach standard [93]. SPMs were generated using a random-effects multiple regression analysis. The predictors were obtained from a box-car design (i.e., a value of 1 for the motion display and 0 for the static display) convolved with a canonical gamma model of the hemodynamic response [94].

Activity in the dynamic display condition was contrasted with that in the static display condition, with the threshold for controlling for false positives among activated voxels set at the false discovery rate (FDR) of q(FDR)<.05 [95]. ROIs were defined from the resulting activation foci (maximum ROI volume was set at 16 mm^3^). We evaluated the sensitivity of the group-defined ROI approach by comparing the group-defined MT^+^ ROI to an MT^+^ ROI defined as the most activated voxel in each individual subject. The HRF dynamics of the group average were similar in the group-defined and individually-defined ROI. Left and right mesial ROIs (SMA, pre-SMA) were averaged together to improve SNR. In order to functionally isolate the primary motor cortex (M1) from surrounding motor cortex, this ROI was defined with the slow-event related task (see below) instead of the localizer task. Because the primary motor cortex responds more to contralateral than to ipsilateral responses [96], M1 could be isolated by contrasting activity between left and right manual responses in the event-related experiment. This contrast yielded activation patterns in left and right precentral gyrus. The M1 ROI was further anatomically restricted by selecting the subset of activated voxels whose locations matched those previously associated with M1[96], [97]. Importantly, this functional definition of M1 does not bias the main speed vs accuracy analysis, as both speed and accuracy trials were collapsed for the purpose of isolating M1. The results presented in **Figure 6** are for right M1 to both ipsi- and contra-lateral manual responses, but comparable results were also obtained when analysis was confined to contra-lateral response trials or to the left M1.

### Slow Event-Related Task

#### Subjects

Thirteen volunteers (age range 20–31, 4 females) participated in this study for pay. Eleven of the 13 volunteers performed the Localizer task.

#### Task design & procedure

The stimulus display was identical to the motion display in the Localizer task, except that a thin (.06°) red or green frame surrounded the 3°×3° square. A trial began when the frame turned from red to green. Participants were instructed to press a left button with their left index finger when they detected leftward motion or the right button with their right index finger when they detect rightward motion. The frame turned from green to red as soon as subjects made a left or right response, or 19.5 s after trial onset if subjects did not make a response during the trial. If motion coherence was not detected, subjects were instructed to withhold responding. However, to equate motor response demands across all trials, subjects were also required to ‘guess’ a response when the frame turned red at the end of trials for which they failed to detect motion coherence. The reaction times of these guesses were not included in any analysis. For all trials, the red frame contained only random motion and stayed on for 13.5 s before it turned to green again, announcing the onset of the next trial.

Trials were presented in blocks (7 trials/block), with the two block types (speed or accuracy) alternating within a run. At block onsets, subjects were visually instructed to emphasize either the speed or accuracy of their responses by showing a display with the letters ‘SPD’ or ‘ACC’ for 3 s, followed by 3 s of fixation and by 6 s of red-framed random motion before the onset of the first trial. In the speed condition, participants were instructed to “respond as quickly as possible as soon as you know the answer”. Subjects were given visual feedback at the end of each fMRI run instructing them to respond more quickly in the next speed blocks if their mean RT (for all speed blocks within the run) was greater than 9 s or if their mean RT was less than 2 seconds faster than their mean RT for accuracy blocks. Participants were not explicitly made aware of the 9 s deadline. For the accuracy condition, subjects were instructed to “Take as much time as needed to make the correct response. Make as few mistakes as possible.” Subjects were given feedback instructing them to be more accurate, at the end of the run, if they made one or more mistakes regarding the direction of coherent motion. Participants were informed that the feedback was not based on performance in trials without motion coherence (baseline trials, see below). Three blocks of trials (alternating between the SPD-ACC-SPD and SPD-ACC-SPD orders across runs to counterbalance any potential influence in linear drift) were presented per fMRI run, and subjects performed 4 to 5 of such runs in an fMRI session.

The experiment included three trial types per speed-accuracy condition. In the coherence trials (3/7 of all trials), a subset of the dots started moving coherently to the left or right, with the strength of this coherence increasing at a rate of 1% per second. The onset of motion coherence began either 0 s, 4.5 s, or 9 s after trial onset. The baseline trials (2/7 of all trials) contained only random motion throughout the trial. The third type of trial (2/7 of all trials) included motion coherence that always began at trial onset and increased at a rate of 2%/second. The sole purpose of the latter trials was to encourage subjects to monitor for motion coherence soon after trial onset. These trials were infrequent, and were therefore not analyzed in the present study. Random intermixing of the three trial types ensured that subjects could not predict if, and when, motion coherence increased. In addition, subjects were told that a trial may or may not contain motion coherence, and that in those that contained motion coherence, this coherence level could begin to increase at any time during the trial. After being given task instructions, participants were presented with one block of practice trials each for the speed and accuracy conditions in the scanner prior to the functional scans.

#### fMRI parameters and analysis

The slow-event fMRI data was acquired and pre-processed as described in the localizer experiment with the exception that the data were not spatially or temporally smoothed. ROIs defined in the localizer task were probed in the slow event-related experiment using custom software programmed in Matlab (The Mathworks Inc, Natick, MA). Time series were extracted from each subject's ROIs and assessed for the presence of spikes (i.e., changes in raw MR activity that exceed the mean of the run by 4 or more SD units). Spikes were replaced with the average value of the immediate neighboring values. Percent signal change was calculated using the volume acquired at trial onset as baseline. Trials with peak activation greater than 3 SDs away from the mean, or with reaction times less than 200 ms were discarded. These *a priori* criteria removed at most 19% of all trials per ROI.

The hemodynamic time courses of the coherence trials were either stimulus- or response-locked. Stimulus locking was performed by aligning and normalizing time courses to the volume corresponding to the onset of motion coherence at either 0 s, 4.5 s, or 9 s. The resulting time courses were then averaged across subjects (random effects analysis). Time courses were also response-locked by aligning all coherence trials to the volume at which the response was made, and normalized to the volume containing the onset of motion coherence. To assess activity build-up prior to the response, we averaged the percent signal change for the volume acquired at response time and the volume immediately preceding it. Subsequent volumes were not analyzed to avoid contamination from activity associated with post-decision processes, such as motor response. Pre-response build-up of activity was then measured by comparing percent signal change for these averaged volumes to the normalized coherence onset. We also carried out paired t-tests between the averaged pre-response and response volumes of the speed and accuracy conditions to determine whether there was differential build-up of activity in the two instruction sets. According to computational models of the SAT [31]–[34], brain regions involved in the SAT should not only demonstrate accumulation of evidence in both speed and accuracy conditions, but also differential accumulation of evidence under both these conditions. Therefore, to be considered candidate neural substrates for the SAT in decision-making, ROIs had to 1) demonstrate activity increase relative to baseline in both speed and accuracy conditions, and 2) exhibit a differential activity increase between these two conditions.

Baseline trials were time-locked and normalized to trial onset. Differences in baseline activity between speed and accuracy conditions were statistically assessed during the time of maximal speed-accuracy difference, corresponding to the average of volumes between 3 s and 19.5 s. We also assessed whether the activity difference between the speed and accuracy conditions in the baseline trials is offset by the difference in the coherence trials by adding up the mean baseline activity to the coherence time courses. Specifically, baseline+coherence time courses were computed by identifying in each subject the mean activation at 4.5 s and 9 s of the stimulus-locked baseline trials. These values were then added to the values of the coherence response-locked data of each corresponding delay conditions. Lastly, the data across all delay conditions (0 s, 4.5 s, and 9 s) were collated for each subject and averaged across subjects.

#### Behavioral data analysis

Subjects made one of three choices in each trial. They decided that either motion coherence was to the right, to the left, or that there was no motion coherence. Our manipulation of speed-accuracy instructions was expected to primarily affect the detection of motion coherence rather than the discrimination of motion direction (i.e., left vs. right). The bias introduced by asking subjects to respond sooner in the speed condition should result in them basing their detection/discrimination decision on lower motion coherence levels, but it should not introduce bias in a choice for the direction of motion coherence. Consistent with this notion, emphasizing response speed increased the probability of responding (falsely) to random motion fluctuations (*p*<.05), but did not bias motion discrimination (*p* = 0.9). Accordingly, the most appropriate behavioral assessment of the effect of speed-accuracy instructions in our experiment is to examine speed-accuracy differences in performance across conditions that differ in the presence or absence of motion coherence. For this reason, *d'* was used to measure sensitivity to detect motion coherence and *c* was used to measure the decision criterion [98]. The use of the signal detection theory (SDT) criterion metric *c* in the present context is further justified by the finding that sequential sampling models of SDT can account for speed accuracy trade-offs [54]. To assess whether these different measures of performance can be tied to specific neural processes, we correlated for the coherence trials the accuracy-speed differences in d' and *c* with the accuracy-speed differences in brain activity (importantly, *d'* and *c* were uncorrelated with one another, *r* = −0.09, *p* = 0.78). Only the data for *c* are presented in the Results section, as the *d'* measure failed to positively correlate with any brain activity, consistent with a prior study [99]. Such null results may be accounted for by the hypothesis that one only needs small changes in neural activity that may be imperceptible to fMRI to produce substantial shifts in *d'* [17].

Although signal detection measures of performance were primarily used for the analysis of motion detection, an SAT was also evident when accuracy performance in motion discrimination was measured (*M* = 99.0% for accuracy, *M* = 95.1% for speed, *t*(12) = 2.19, *p*<0.05). Performance in motion discrimination was maintained at a high level in order to ensure that a sufficient number of correct trials were available for fMRI data analysis. It has been shown that the coupling between speed and accuracy still holds even at high accuracy levels [40].

## Supporting Information

Table S1 Activation t-values from the Coherence Trial Analysis.
(0.10 MB DOC)

Table S2 Activation t-values from the Baseline Trial Analysis.
(0.10 MB DOC)

Figure S1 Hemodynamic time courses for hits (motion coherence trials with a correct response in red) and false alarms (baseline trials with an early, erroneous response in cyan) in the (A) pLPFC and (B) pre-SMA. One participant made only one false alarm in the speed condition and was removed from the analysis. All trials were response-locked (R) and normalized to the onset of the trial. Only hits and false alarms are shown for the speed condition because there were too few false alarms in the accuracy condition.
(0.35 MB TIF)
