# Supplementary material for: fMRI Evidence for a Dual Process Account of the Speed-Accuracy Tradeoff in Decision-Making
Source: PLoS One. 2008 Jul 9;3(7):e2635. doi: 10.1371/journal.pone.0002635 (PMC2440815; doi:10.1371/journal.pone.0002635)
Supplement: Table S1 — Activation t-values from the Coherence Trial Analysis. (0.10 MB DOC) [file pone.0002635.s001.doc]

**Table S1. Activation t-values from the Coherence Trial Analysis.**

| Region |  | Left |  |  |  | Right |  |
| --- | --- | --- | --- | --- | --- | --- | --- |
|  | SPD | ACC | diff. |  | SPD | ACC | diff. |
| Superior Frontal Gyrus | -0.89 | -0.14 | 0.73 |  |  |  |  |
| Precentral Gyrus | -0.43 | 0.08 | 0.53 |  | -0.24 | -0.31 | -0.09 |
| Precentral Sulcus | 1.45 | -0.32 | -0.65 |  |  |  |  |
| S1 and M1 | -0.95 | -1.07 | 0.12 |  | -0.53 | 0.24 | 0.42 |
| Pre-SMA | 2.05 | 3.76** | 2.28* |  | 1.61 | 2.29* | 1.79 |
| SMA | 0.56 | 0.91 | 0.76 |  | 0.08 | 1.93 | 2.27* |
| Anterior Cingulate (BA 24) | -0.47 | 0.43 | -1.23 |  | -0.17 | -0.47 | -0.61 |
| Anterior Cingulate (BA 32) | 0.44 | 0.95 | 0.45 |  | 0.57 | 0.13 | -0.49 |
| Anterior IPS |  |  |  |  | -0.77 | 1.09 | 1.61 |
| DIPSA | -0.33 | 1.06 | 1.75 |  | -0.60 | 1.52 | 1.82 |
| dPM | 4.10** | 1.11 | -0.37 |  | 4.48*** | 4.90*** | 0.57 |
| Postcentral Gyrus | 1.03 | 0.31 | 0.02 |  | 0.30 | 3.23** | 2.57* |
|  | 0.37 | 0.32 | 0.11 |  | -0.47 | 0.27 | 0.70 |
| vIPL | -0.23 | 0.57 | 1.69 |  | 1.08 | 4.28** | 2.20* |
| vPM | 2.10 | 2.40* | 1.00 |  | 3.66** | 0.69 | -1.47 |
| pLPFC | 2.81* | 1.79 | -0.46 |  | 3.42** | 4.99*** | 2.23* |
| TPJ | -0.37 | -0.80 | -1.02 |  | 0.30 | 0.67 | 0.51 |
| Thalamus | 1.05 | -0.79 | -1.56 |  | 0.86 | 2.63* | 0.33 |
| Insula | -0.38 | 1.22 | 1.85 |  | -0.62 | 0.72 | 1.73 |
| Putamen | 0.68 | 2.93* | 1.76 |  | 0.84 | 1.41 | 0.23 |
| Extrastriate | 0.33 | -0.74 | -0.99 |  | 0.33 | -0.61 | -0.69 |
| Superior Colliculus | 1.96 | -0.97 | -1.77 |  |  |  |  |
| Anterior Insula | 0.11 | 2.64* | 1.18 |  | 2.47* | 4.57*** | 1.20 |
| MT+ | 1.99 | 0.38 | -1.18 |  | 5.75*** | 3.71** | 0.23 |
| Anterior IFG | 1.34 | 0.06 | -0.99 |  |  |  |  |
| Anterior Cerebellum | -2.41* | -1.97 | -1.36 |  | -2.01 | -2.67* | -2.03 |
| Posterior Cerebellum | -0.40 | -0.37 | -0.04 |  | -1.57 | -2.05 | -0.64 |

Notes:

The speed (SPD) and accuracy (ACC) analyses reflect whether the average of the response volume and the pre-response volume were significantly different from zero. The paired t-values of the difference (diff; ACC-SPD) of these conditions is also provided.

*** p<0.001

**p<0.01

* p<0.05
