# Supplementary material for: fMRI Evidence for a Dual Process Account of the Speed-Accuracy Tradeoff in Decision-Making
Source: PLoS One. 2008 Jul 9;3(7):e2635. doi: 10.1371/journal.pone.0002635 (PMC2440815; doi:10.1371/journal.pone.0002635)
Supplement: Table S2 — Activation t-values from the Baseline Trial Analysis. (0.10 MB DOC) [file pone.0002635.s002.doc]

**Table S2.** Activation t-values from the Baseline Trial Analysis.

| Region |  | Left |  |  |  | Right |  |
| --- | --- | --- | --- | --- | --- | --- | --- |
|  | SPD | ACC | diff. |  | SPD | ACC | diff. |
| Superior Frontal Gyrus | 0.42 | 1.68 | -0.54 |  |  |  |  |
| Precentral Gyrus | 0.14 | 0.51 | -0.83 |  | 0.58 | -0.23 | -1.05 |
| Precentral Sulcus | 1.16 | 1.96 | 0.31 |  |  |  |  |
| S1 and M1 | -0.39 | -1.38 | -1.01 |  | -0.25 | -0.22 | -.50 |
| Pre-SMA | 3.72** | 3.21** | -2.50* |  | 3.30** | 3.52** | -2.80* |
| SMA | 2.19* | 1.74 | -2.03 |  | 2.12 | 1.39 | -1.82 |
| Anterior Cingulate (BA 24) | -1.05 | -0.59 | -0.53 |  | -0.25 | 0.14 | -1.45 |
| Anterior Cingulate (BA 32) | 1.39 | 1.00 | -1.31 |  | 1.39 | 1.53 | -1.34 |
| Anterior IPS |  |  |  |  | 1.07 | 2.16 | -0.18 |
| DIPSA | -0.19 | -0.27 | -0.57 |  | 1.14 | 1.39 | -1.24 |
| dPM | 4.23** | 3.69** | -1.96 |  | 3.39** | 3.96** | -1.14 |
| Postcentral Gyrus | -0.13 | 1.11 | 0.14 |  | 0.82 | 1.58 | -0.32 |
|  | 0.82 | 0.74 | -2.69* |  | 2.35* | 1.18 | -2.95* |
| vIPL | 0.80 | 0.75 | -0.94 |  | 2.88* | 3.45** | -0.58 |
| vPM | 1.79 | 1.97 | -1.39 |  | 3.41** | 3.14** | -1.46 |
| pLPFC | 1.05 | 1.89 | -1.95 |  | 4.57** | 4.80*** | -2.27* |
| TPJ | 1.19 | -0.04 | -1.57 |  | 1.80 | 1.16 | -2.35* |
| Thalamus | 1.76 | 0.22 | -1.91 |  | 4.10** | 1.24 | -2.70* |
| Insula | 0.28 | -0.93 | -2.12 |  | 0.19 | -0.27 | -1.98 |
| Putamen | 2.26* | 1.71 | -2.04 |  | 3.27** | 0.37 | -2.07 |
| Extrastriate | 1.54 | 2.42 | 0.10 |  | 2.89* | 2.27* | -1.09 |
| Superior Colliculus | 2.52* | 0.46 | -1.11 |  |  |  |  |
| Anterior Insula | 2.08 | 0.63 | -3.18** |  | 5.47*** | 3.82** | -5.65*** |
| MT+ | 2.01 | 0.80 | -1.78 |  | 8.38*** | 6.46*** | -1.84 |
| Anterior IFG | 0.69 | -1.65 | -1.15 |  |  |  |  |
| Anterior Cerebellum | 0.26 | -2.25* | -1.82 |  | 0.11 | -3.24** | -2.59* |
| Posterior Cerebellum | 1.87 | -0.55 | -0.80 |  | 1.42 | -0.43 | -1.43 |

Notes:

The ACC-SPD difference (diff.) reflects accuracy and speed differences of the summed intermediary volumes, from 9s to 15s after the trial onset. The SPD and ACC analyses reflect a one-sample t-test of the averaged activity from the volumes at the time of the trial and the volume that immediately precedes the end of the trial.

*** p<0.001

**p<0.01

* p<0.05
