# Supplementary figures and images for: fMRI Evidence for a Dual Process Account of the Speed-Accuracy Tradeoff in Decision-Making
Source: PLoS One. 2008 Jul 9;3(7):e2635. doi: 10.1371/journal.pone.0002635 (PMC2440815; doi:10.1371/journal.pone.0002635)

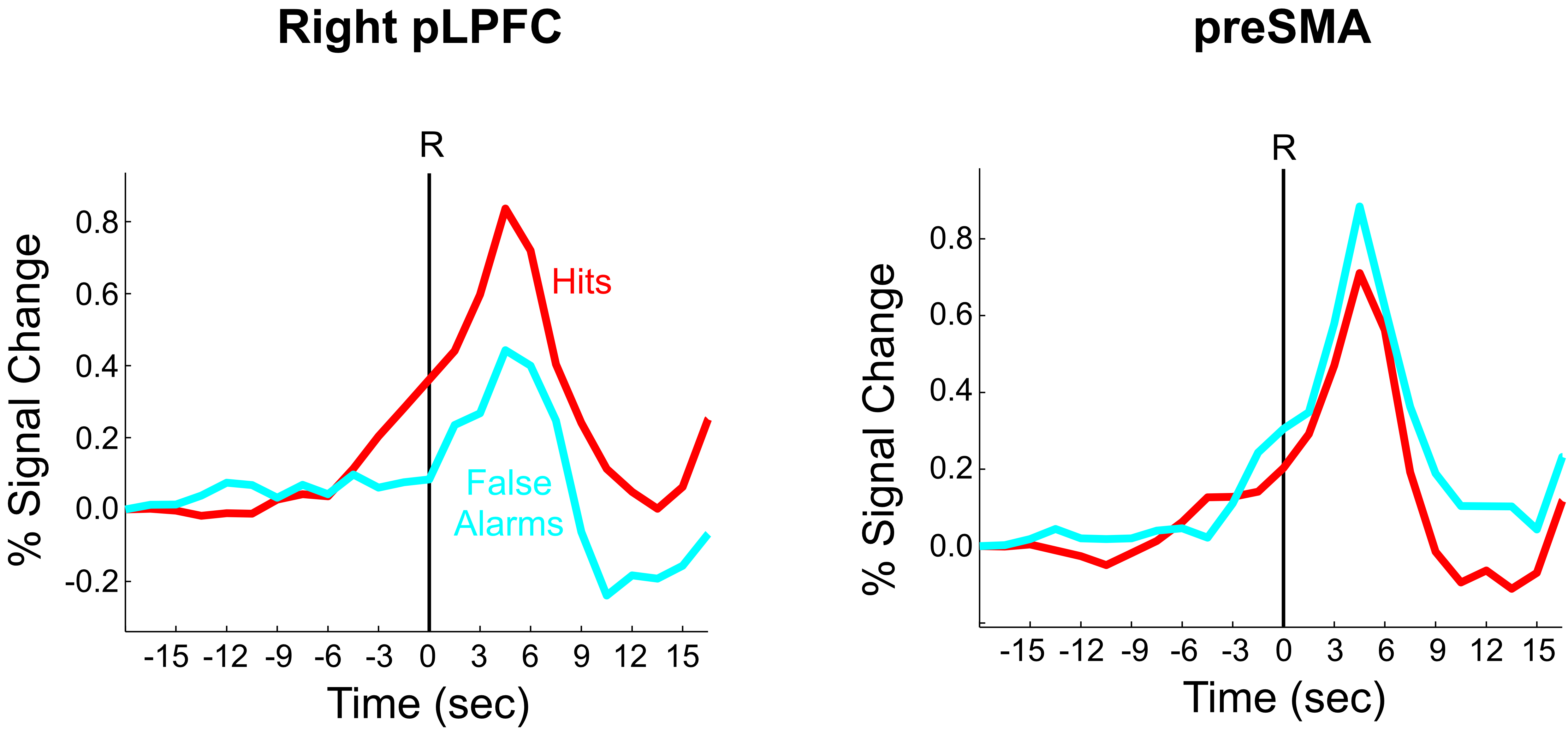

Supplement: Figure S1 — Hemodynamic time courses for hits (motion coherence trials with a correct response in red) and false alarms (baseline trials with an early, erroneous response in cyan) in the (A) pLPFC and (B) pre-SMA. One participant made only one false alarm in the speed condition and was removed from the analysis. All trials were response-locked (R) and normalized to the onset of the trial. Only hits and false alarms are shown for the speed condition because there were too few false alarms in the accuracy condition. (0.35 MB TIF) [file pone.0002635.s003.tif]
